# In Vivo Stability of Polyurethane-Based Electrospun Vascular Grafts in Terms of Chemistry and Mechanics

**DOI:** 10.3390/polym12040845

**Published:** 2020-04-07

**Authors:** Alexander A. Gostev, Inna K. Shundrina, Vitaliy I. Pastukhov, Alexey V. Shutov, Vera S. Chernonosova, Andrey A. Karpenko, Pavel P. Laktionov

**Affiliations:** 1 Meshalkin National Medical Research Center, Ministry of Health of the Russian Federation, 630055 Novosibirsk, Russia; andreikarpenko@rambler.ru (A.A.K.); lakt@niboch.nsc.ru (P.P.L.); 2 Vorozhtsov Novosibirsk Institute of Organic Chemistry, Siberian Branch, Russian Academy of Sciences, 630090 Novosibirsk, Russia; i.shundrina@nsu.ru (I.K.S.); v.pastukhov@nsu.ru (V.I.P.); 3 Novosibirsk State University, ul. Pirogova, 2, 630090 Novosibirsk, Russia; a.shutov@g.nsu.ru; 4 Institute of Chemical Biology and Fundamental Medicine, Siberian Branch, Russian Academy of Sciences, 630090 Novosibirsk, Russia; vera_mal@niboch.nsc.ru; 5 Lavrentiev Institute of Hydrodynamics, Siberian Branch, Russian Academy of Sciences, 630090 Novosibirsk, Russia

**Keywords:** tecoflex, pellethane, gelatin, electrospinning, vascular grafts, polyurethane stability in vivo

## Abstract

The biostability of the polyurethanes Tecoflex EG-80A and Pellethane 2363-80A, used as basic polymers of the vascular grafts (VGs) produced by electrospinning, as well as the tensile strength of Tecoflex VGs, are studied. Solutions of Tecoflex or Pellethane with gelatin and bivalirudin in 1,1,1,3,3,3-hexafluoroisopropanol are used for VG production. After 1, 12, and 24 weeks of VG implantation in the infrarenal position of the abdominal aorta of Wistar rats, VGs are explanted, fixed in formalin, freed from outer tissues, dialyzed, and dried. The polyurethanes are extracted from VGs by dispersion/extraction in tetrahydrofuran (THF) and freed from the excess of THF-insoluble biopolymers. The stability of polyurethanes is assessed by IR spectroscopy and gel permeation chromatography. Pellethane has emerged to be stable at all experimental points. Tecoflex loses approximately 10% of its molecular weight (both *M*_n_ and *M*_w_) after 3 months and restored its initial value within 6 months of its functioning as a graft. Mechanical testing demonstrates a 30% reduction in the tensile strength after 3 months in VG and a 10% increase after 6 months. The stability and mechanical properties of polyurethane-based VGs demonstrate their utility for the reconstitution of damaged arteries.

## 1. Introduction

Polyurethanes are a class of polymers with a wide range of properties, which makes them promising for medical purposes. They are used for manufacturing both manifold medical instruments [1] and grafts [2], and may have different biodegradation rates depending on the structures of their soft and hard segments [3,4]. As is known, polyurethanes are biodegraded via hydrolysis of their ester and urethane groups or oxidation of the aliphatic fragments carrying ether bonds [5]. However, the stability of thermoplastic polyurethanes in biological systems and the mechanisms underlying their degradation have been studied for films, sheets, or nonporous monolithic type items manufactured, as a rule, by cast molding [6]. Only a few studies considered the stability of polyurethanes in porous objects [7,8], while these particular materials, including those produced of a mixture of synthetic and natural polymers, are currently regarded as promising for tissue engineering in various organs [9,10,11].

One of the promising methods for the manufacture of materials with a larger specific surface area and porous structure for tissue engineering is electrospinning (ES). This method makes it possible to produce fibers from polymer solution and their mixtures and to fold fibers into 3D matrices of various shapes, different fiber orientations, and anisotropies of mechanical properties. Combining polymers and additional components in the solution for ES, it is possible to produce fibers with different properties. This provides an opportunity to lay up several types of fibers, thus offering unlimited potential for the production of 3D matrices. The electrospun matrices, especially those produced from polymeric mixtures, fundamentally differ from the cast-molded polymeric grafts. These matrices have a large specific surface area and contain molecules of different polymers in each fiber, which are able to interact with each other via the functional groups they carry [12].

Many polyurethanes are fiber-forming polymers, and consequently have found wide use in the ES of vascular [13] and valve grafts [14,15,16] and other tissue-engineered products [17,18]. Since ES allows the fibers to be produced from mixtures of polymers, the problem of insufficient biocompatibility is resolvable by fabricating 3D matrices (or vascular grafts, VGs) of the blends comprising synthetic and natural polymers. Such fibers expose both synthetic and natural polymers on their surface and mimic well the extracellular matrix [19]. In addition, the polymer mixtures make it possible to produce materials with upgraded physical, mechanical, and chemical properties compared with the initial polymers [20].

Depending on the target function, the simulated tissue-engineered construct must be biologically stable (resistant to hydrolysis and enzymes, for example, in the case of valve leaflets) or, on the contrary, biodegradable (for example, the substrate for growing hyaline cartilage cells) and the polymers used for its manufacture must ensure the maintenance of its shape and mechanical properties over a specified time period. Evidently, the biodegradable polymer must be replaced by newly synthesized tissue so that the stability and function of the remodeled tissue region is preserved at a necessary level. When an important fragment of tissue or organ, for example, a region of a blood vessel, is remodeled, the strength of the used construct and its long-term functional stability are the decisive characteristics of such a VG, since its untimely destruction may have a lethal outcome. In earlier papers, we have demonstrated that the 3D matrices produced from protein-enriched polyurethanes possess good mechanical properties, and structural stability in vitro, bio- and hemocompatibilities, and are, thus, valuable materials for the production of small-diameter vascular grafts [19,20,21].

In this paper, we describe biostability of the polyurethanes Tecoflex EG-80A and Pellethane 2363-80A, differing in the structure of their hard segments but similar in their soft segments, within the fibers made of polyurethane–gelatin blends used to produce VGs by ES. The VGs were implanted in the rat abdominal aorta and explanted after 12 or 24 weeks to assess the biostability of these two types of polyurethane molecules; in addition, the tensile strength of explanted VGs made of Tec-80A was estimated. The VGs were produced of the blends of these polymers with gelatin (and a thin inner layer of gelatin and bivalirudin) because we have previously demonstrated that the 3D matrices produced from these blends are well bio- and hemocompatible, stronger than those of pure polyurethanes, and thus are valuable materials for VG production [19,20].

## 2. Materials and Methods

### 2.1. Electrospinning of Vascular Grafts

The ES solutions were prepared from the stock polymer solutions in 1,1,1,3,3,3-hexafluoroisopropanol, HFIP (Sigma, St. Louis, MI, USA) 10% Tecoflex EG-80A, Tec-80A (Lubrizol Advanced Materials, Avon Lake, OH, USA), 10% Pellethane 2363-80A, Pel-80A (Lubrizol Advanced Materials, Avon Lake, OH, USA), 5% gelatin (GL) solution (Sigma, St. Louis, MI, USA, from porcine skin), and 1.5% bivalirudin (BV) solution (Sigma, St. Louis, MI, USA). Pel-80A is insoluble in any solvent and mixtures of solvents but swells in HFIP. To dissolve Pel-80A granules, 0.35 g of Pel-80A were added to the mixture of 10 mL of HFIP and 26 µL of hydrofluoric acid. After 16-h incubation at 37 °C, the acid was neutralized with 65 µL of 10 M sodium hydroxide solution; the insoluble precipitate was removed after centrifuging the solution at 80,000 rpm. The molecular weight of the resulting polymer was estimated from the Pel-80A viscosity at different concentrations measured in a DV-II+ Pro viscometer (Brookfield Engineering Labs, Inc., Middleboro, MA, USA) using Shultz–Blashke and Mark–Kuhn–Houwink equations as described in [22,23].

The ES solutions contained 3% Tec-80A (wt/vol) with 15% GL and 3.5% Pel-80A with 10% GL or the same solutions with 1.5% BV [21]. GL and BV concentrations in matrices are shown as the percentage of polyurethane (wt/wt).

The VGs with a wall of 130–150 µm and diameter of 2 mm were produced using an NF-103 (MECC, Fukuoka, Japan) electrospinning device under the following conditions: feed rate, 1–1.15 mL/h; capillary-collector distance, 19–20 cm; voltage, 18.5–24 kV; rotation speed of collector (diameter, 2.7 mm), 300 rpm; ~80 mm/min nozzle moving and humidity, 25–35%. The VGs had two layers (inner layer of 25–30 µm composed of polyurethane, GL, and BV, and outer layer of 100–105 µm of polyurethane and GL, applied sequentially). Thickness was measured by electronic outside micrometer (0–25 mm, 0.001), Schut Geometrical Metrology, 908.750. The length of tubes was 75–80 mm, but 10–15 mm pieces were implanted in an infrarenal position.

VGs were incubated in 0.05 M NaHCO_3_ (pH 9.1) in a horizontal shaker for 20 min to moisten the material and then treated with 2% glutaraldehyde/0.05 M NaHCO_3_ solution for 2 h at a room temperature. After incubation, the matrices were washed thrice with 0.05 M NaHCO_3_ (pH 9.1) for 5 min. The remaining free reactive groups were blocked by incubating the matrices in 10 mM glycine (pH 9.1) for 30 min followed by incubation in freshly prepared 1 mg/mL NaBH_4_ for 15 min. After the incubation, the matrices were thoroughly washed with three changes of H_2_O and air-dried.

### 2.2. Implanting VGs and Examining Their In Vivo Functioning

This test involved 36 Wistar rats with an average age of 6.8 ± 0.15 months and average weight of 440 ± 40 g (18 rats per polyurethane, six animals per timepoint). All manipulations with animals complied with the European Convention of laboratory animal protection (Rozemond, H., 1986, Laboratory animal protection: The European Convention and the Dutch Act., Veterinary Quarterly, 8 (4), 346–349), meeting all requirements of the WMA Declaration of Helsinki (2000). Before implantation, VGs were sterilized by electron beam irradiation (15 KGy). The animals were kept under stationary vivarium conditions with natural illumination, standard diet, and water ad libitum. For each observation period (1, 12, and 24 weeks), a specimen of each type of experimental VGs was implanted to each individual in the infrarenal aorta; the surgical manipulations and after-surgical maintenance are detailed in [24].

Upon completion of the observation period, the animals were narcotized to explant the VGs with 5 mm of the aorta via the second laparotomy. The animals were then immediately withdrawn from experiment by adding a double dose of narcotic and subsequently decapitated.

### 2.3. VG Treatment after Explanting

The explanted VG was rinsed with physiological saline solution and fixed with 4% formaldehyde for further histological examination. Before assessing the polyurethane stability, VG was washed from formaldehyde with physiological saline solution; a VG fragment with a length of 10 ± 1.2 mm was cut off. The fragment was longitudinally cut; the VG fragments of biological tissue on its outer and inner sides were carefully removed. Then, VGs were individually incubated in seven changes of sterile apyrogenic distilled water (5 mL each) for 1 week at a room temperature. After the completion of dialysis, VGs were air-dried in a sterile laminar air flow for 48 h and weighed with an accuracy of 0.1 mg. The VG fragments in Eppendorf tubes with glass beads and 0.5 mL of tetrahydrofuran (THF) were placed into an Eppendorf 5432 mixer (Eppendorf AG, Hamburg, Germany) and incubated for 7 days until the complete breakup of the VG specimen. The remains of undissolved biological tissue in THF were sedimented by centrifugation at 12,000× *g* for 10 min and the supernatant, containing dissolved polyurethane, was further assayed.

The matrices produced by ES of the same solutions as the VGs were used as the control specimens; part of them was incubated in pyrogenic distilled water for 3 days and dried as described above and the remaining matrices were not subject to any treatment. The control samples and explanted VGs were dissolved in THF for 7 days (as described above). The subsequent processing of the samples was performed as described earlier.

### 2.4. Gel Permeation Chromatography (GPC)

GPC was performed using an Agilent LC 1200 setup equipped with an isocratic pump, a PL-gel 5 µm Mixed-C column PL 1110–6500, and a refractometric detector. The system, operating at 40 °C with an eluent (THF) flow rate of 1 mL/min, was calibrated against narrow polystyrene standards (ranging from 162 to 6,035,000 g mol^−1^).

### 2.5. Fourier-Transform Infrared Spectroscopy (FTIR)

The FTIR spectra of polyurethanes were recorded in a Tensor 27 spectrometer. FTIR scans were collected on the completely dried thin films cast on potassium bromide disks from HFIP solution. The spectra covered the infrared region of 4000–500 cm^−1^.

### 2.6. Testing Tensile Strength

The VGs implanted to animals and the explanted VGs (freed of external tissues) were cut into segments with a length of 10 mm. Each segment was longitudinally cut into halves to get two bands of 10 × 1.2 mm. The edges of wet bands (approximately 2 mm) were fixed with microclamps to record the diagram of wet VG stretching (along the VG axis or the blood flow) using a Zwick/Roell Z10 (Zwick Roell Group, Ulm, Germany) universal testing machine, as described in ISO 7198:1998 [25], under constant wetting with a water spray system. The VG samples of at least two animals were tested for each VG type.

### 2.7. Statistical Analysis

The data are given as the mean ± standard deviation. The statistical significance of the difference between groups was determined with the help of the McNemar’s test. The probability values less than 0.05 were considered significant.

## 3. Results and Discussion

As is known, the addition of protein to the solutions for ES not only provides a long-term exposure of the protein to the surface of fibers [12], but also increases the strength of the produced materials [20,26]. The materials with an implicit fiber orientation were obtained under the described ES conditions [20]. The exposure of GL on the surface of the 3D matrices produced by the ES of polyurethanes ensures that human primary endotheliocytes properly attach to these matrices and proliferate, whereas BV reduces blood cell adhesion [21]. Since poor VG endothelization is one of the causes leading to the formation of neointima, clotting, and stenosing [27,28], we used the VGs produced of Tec–GL with an inner layer of Tec–GL–BV [28] for implantation in animals.

Pel-80A (Lubrizol, Inc., United States) was insoluble in any of the recommended solvents and its treatment according to the earlier described procedure [29] did not allow for dissolution in organic solvents. Pel-80A is produced with a slight excess of diisocyanate [30]. The isocyanate terminal groups can react with urethane groups, forming allophonate cross-links, and thereby decreasing the solubility of polymer. To destroy the cross-links and obtain a soluble polymer, we utilized a partial hydrolysis of Pel-80A with hydrofluoric acid. The molecular weight of the resulting polymer was determined by measuring the viscosity of the polymer solution of different concentrations. The characteristic viscosity was determined using the Shultz–Blashke equation after measuring the viscosities of the solutions and solvent. Then, the molecular weight of hydrolyzed Pel-80A was calculated using the Mark–Kuhn–Houwink equation and the reference values of coefficients *k* and α from the Shultz–Blashke equation. Since the viscosity of the 0.035 g/L Pel-80A solution in HFIP was 140 cP, the viscosity average molecular weight (*M*_v_) of the polymer varied in the range of 110–130 kDa [31,32].

The average molecular weight (*M*_n_) and weight average molecular weight (*M*_w_) determined using size exclusion chromatography were 105 and 190 kDa, respectively (Table 1). Note that the Pel-80A molecular weight after hydrolysis remained higher as compared with the Tec-80A molecular weight, which was not hydrolyzed.

**Figure 1:**
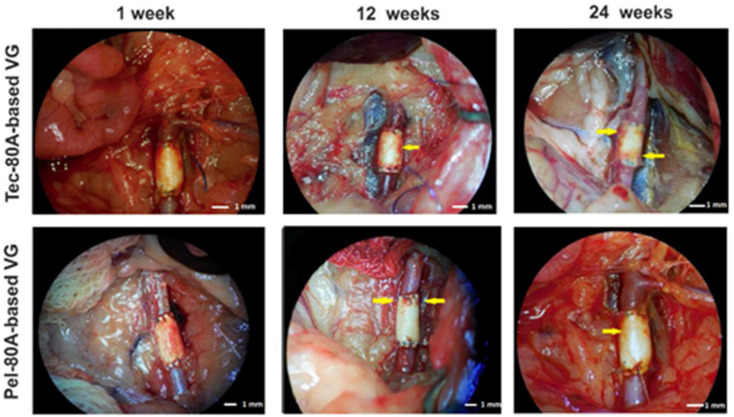
The vascular graft (VG) view during explantation at different points of observation (Carl Zeiss OPMI Pico surgical microscope). The arrows demonstrate the ingrowth of tissues from the outer VG side.

All VGs were permeable according to the data of intravital ultrasound scanning. After explanting, the formation of neointima and ingrowth of tissues from the outer VG side were observable in some sites of VGs (Figure 1); correspondingly, these tissue fragments were removed to decrease the contamination of polyurethanes with biological molecules and biopolymers. Tissues’ ingrowth process indicates that the electrospun matrix performs a scaffold function for normal autologous tissue.

The preliminary experiments have shown that THF is the most suitable solvent for extracting the polyurethanes from their mixture with protein because proteins are insoluble in it (unlike DMSO, DMF, and, especially, HFIP). Although polyurethanes are readily soluble in THF, only part (approximately 25–30%) of the polyurethanes from the polyurethane–GL matrices dissolved in this solvent. This pattern is observable for both the VGs explanted from rats and freshly produced matrices or the matrices contacting water (hydration and subsequent drying have no effect on the polyurethane dissolution from the ES matrices). We have earlier demonstrated using x-ray photoelectron spectroscopy and small-angle x-ray scattering that polycaprolactone and human serum albumin molecules interact when producing the matrices of these compounds [12]. The 3D matrices produced by ES from Tec-80A-gelatin blend have similar properties and almost do not leave GL [19,20]; most likely, a similar process takes place in these materials as well.

**Figure 2:**
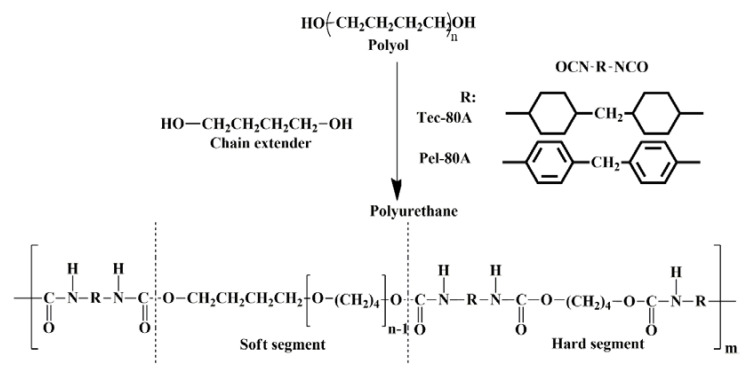
Structures of Tec-80A and Pel-80A polyurethanes.

The polyurethanes extracted from matrices using the described approach were analyzed by GPC and IR Fourier transform spectroscopy. The chemical structure of polyurethanes is the key factor that determines their stability [33,34,35]. Tec-80A is synthesized based on poly(tetramethyleneoxide), aliphatic hydrogenated 4,4′^−^methylenediphenyl diisocyanate, and chain extender 1,4-butanediol. Unlike Tec-80A, Pel-80A is synthesized utilizing aromatic 4,4′^−^methylenediphenyl diisocyanate. Thus, the polyurethanes Tec-80A and Pel-80A have the soft segments of the same structure but differ in their hard segments (Figure 2).

Several researchers believe that the absence of soft segments with ester groups in the structure of the selected polyurethanes makes them sufficiently stable towards the hydrolysis, affecting soft segments [33]. The degradation of the polyurethanes carrying ether groups in their soft segments, Tec-80A and Pel-80A included, in biological media may be associated with the hydrolysis of urethane groups and oxidation of aliphatic segments, carrying ether bonds [31]. As a rule, the reactions of hydrolysis decrease the molecular weight of polyurethanes. The oxidative degradation of polyurethanes in biological media is determined by the oxidation of the aliphatic segments carrying ether bonds caused by various oxidants (oxygen or transition metals), which can be generated by cells (for example, superoxide radical in macrophages, peroxide radical degradation by peroxidase or catalase, and so on) [36,37]. The oxidation can cause a decrease in molecular weight, the accumulation of oxidized fragments in the structure of polymer, and the formation of new intermolecular bonds and cross-links [34,38].

The molecular weight characteristics of the polyurethanes extracted from matrices before and after implantation are listed in Table 1. These characteristics of Pel-80A did not change after 1, 12, and 24 weeks in the rat body. The molecular weight of Tec-80A insignificantly changed and did not correlate with the time it stayed in the rat body. However, the dispersity increased while being implanted, thereby suggesting random hydrolysis.

Thus, the polyurethanes Tec-80A and Pel-80A within protein-enriched 3D matrices (polyurethane-GL) fabricated by ES of the polyurethane solutions with GL display a good biostability for 6 months after in vivo implanting at least in the VGs implanted in the rat infrarenal aorta. Pel-80A, carrying aromatic fragments in its hard segments, avoided hydrolytic and oxidative degradation and displayed a higher stability as compared with Tec-80A, an aliphatic polyurethane, which demonstrates weak signs of degradation after a long-term implantation in the living body.

IR spectroscopy was used for analysis of the oxidative and hydrolytic degradations of polyurethanes. The main absorption bands in the IR spectrum used for assessing the biostability of polyurethanes are 1730 cm^−1^ (hydrogen-bonded urethane carbonyl, NH–CO–O–), 1703 cm^−1^ (nonhydrogen-bonded urethane carbonyl, NH–CO–O–), 1220 cm^−1^ (urethane C–N, Amide III), 1110 cm^−1^ (ether C–O–C in the soft segment), and 1075 cm^−1^ (urethane ether C–O–C) [39,40].

The oxidation of soft segments influences the changes in the intensity of the absorption band at 1110 cm^−1^, while the hydrolysis of urethane bonds affects the changes in the intensity of the bands at 1220, 1075, 1703, and 1730 cm^−1^ [39]. The ratio of intensities of absorption bands is used for the comparative analysis of polyurethane degradation (Table 2). Note that any new absorption bands were unobservable in the IR spectra of the polyurethanes explanted from rats as compared with the control samples.

Tec-80A displayed a decrease in the ratios of absorption intensities *I*_1110_/*I*_1220_ and *I*_1110_/*I*_1075_ with an increase in the time this VG stayed in the rat body versus Pel-80A, which did not display any noticeable changes in the ratios of these absorption bands up to 24 weeks of observation. Despite the same structure of the soft segments, their oxidation in Tec-80A was more rapid than Pel-80A. the different stabilities towards oxidative degradation can be associated with two main factors. First, the structure of the hard segments and their content also influences the biostability of polyurethanes. The hard segments of Pel-80A contain aromatic fragments of 4,4′-methylenediphenyl diisocyanate, whereas the hard segments of Tec-80A are completely aliphatic. Several researchers report that the polyurethanes involving aromatic diisocyanates are more stable as compared with their aliphatic analogs [30].

Second, the exposition of GL on the surface of matrices shown earlier [19,20] can also influence the polyurethane oxidation. The matrices are mainly oxidized on their surface and the more GL molecules exposed on the surface, the less the polyurethane oxidation. A change in the ratio of intensities *I*_1075_/*I*_1220_ in IR spectra suggests an insignificant hydrolysis of the urethane bonds in Tec-80A.

**Figure 3:**
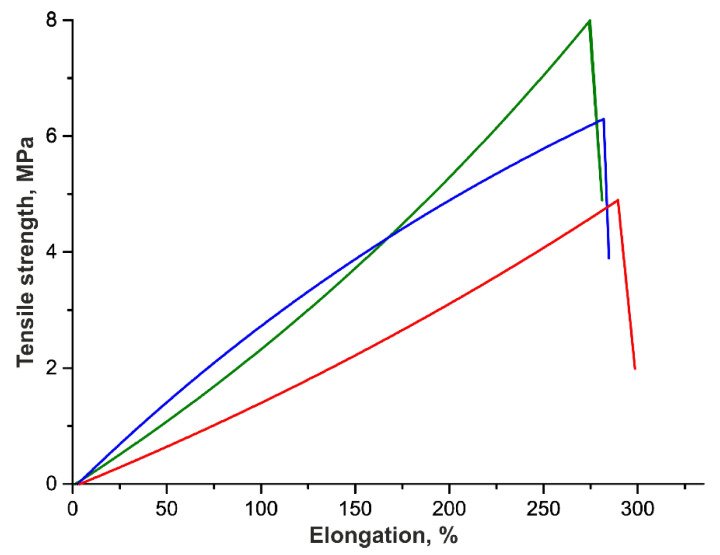
Typical tensile stress diagrams of unimplanted VGs made of Tec-80A (blue line) and the same VGs 3 months (red line) and 6 months (green line) after implantation in rats. Plots are shown as the mean of VG strength measured in three animals.

The tensile strength of VGs was measured to estimate the effect of the implantation period and the change in Tec-80A molecular weight on the strength (Table 3, Figure 3). As is seen, the VG strength drops by approximately 28% after a 3-month functioning in the rat infrarenal aorta. However, the VG strength was restored by the end of 6 months and even increased by 14% as compared with the initial grafts. Note that all VGs display approximately the same elongation at break and almost do not differ in this characteristic from the initial VGs after 3-month implantation, but become more stiff and strong after 6 months. Presumably, the contact between the fibers of the polymer is disturbed in the initial period (3 months), retaining a stiffness close to its initial values; however, de novo synthesized tissue grows into the graft, thereby decreasing the mobility of fibers relative to each other and increasing the strength and stiffness of VG wall after 6 months. This assumption is confirmed by other data on the mechanical characteristics of the electrospun polyurethane-based VGs after their implanting in the rat infrarenal aorta by days 180 and 365 after surgery [36]. As in our case, the authors observed an increase in the VG strength by 20% on day 180 as compared with the unimplanted graft [41].

Note in conclusion that part of the polyurethane that is insoluble in THF is in the complex with biopolymers. In our work, we have not succeeded in separating the polyurethane–GL complexes after the ES of the fibers by either extraction/chromatography or the protease treatment of matrices. Most likely, the stability of polyurethanes in the complexes with biopolymer can differ; however, this requires a separate study.

## 4. Conclusions

Thus, the stability of the polyurethanes Tecoflex EG-80A and Pellethane 2363-80A within the electrospun vascular grafts produced from blends with gelatin has been studied. It is shown that Pellethane 2363-80A molecules are stable for at least 6 months, while Tecoflex EG-80A molecules display certain changes in their average molecular weight 3 months after implantation. However, this change does not influence the tensile strength of the vascular grafts explanted after 6 months. The tensile stress diagrams of the vascular grafts explanted after 3 and 6 months show that the interaction of the grafts with cells and body fluids influences their strength/elasticity. The impact of de-novo-formed connective tissue on the mechanical properties of ES-produced PU vascular grafts undoubtedly needs to be studied, but it is not a simple task (a set of connective tissue proteins as well as their orientation must be studied) and demands a delicate separate study.

## Figures and Tables

**Table 1:** Molecular weight characteristics (*M*_n_, average molecular weight; *M*_w_, weight average molecular weight; and *n*, dispersity) of the polyurethanes extracted from vascular grafts explanted from rats.

No.	Composition of Matrix	*M*_n_, kDa	*M*_w_, kDa	*n*
1	Tec-80A control	102 ± 2	152 ± 4	1.49 ± 0.02
2	Tec-80A (1 week after implantation)	90 ± 2	137 ± 3	1.52 ± 0.04
3	Tec-80A (12 weeks after implantation)	81 ± 3	130 ± 5	1.60 ± 0.02
4	Tec-80A (24 weeks after implantation)	91 ± 3	138 ± 5	2.13 ± 0.01
*p*		0.02	0.04	0.24
5	Pel-80A control	105 ± 4	190 ± 6	1.80 ± 0.04
6	Pel-80A (1 week after implantation)	105 ± 6	190 ± 7	1.80 ± 0.05
7	Pel-80A (12 weeks after implantation)	115 ± 5	190 ± 3	1.65 ± 0.04
9	Pel-80A (24 weeks after implantation)	110 ± 3	194 ± 5	1.76 ± 0.01
*p*		0.11	0.79	0.06

The data are shown as the mean value for three biological donors ± standard deviation.

**Table 2:** Dependence of the ratio of absorption band intensities in the IR Fourier transform spectra of polyurethanes on the time of vascular graft functioning in infrarenal position.

Polyurethane in Matrix	Time after Explantation	*I*_1703_/*I*_1730_	*I*_1110_/*I*_1220_	*I*_1075_/*I*_1220_	*I*_1110_/*I*_1075_
Tec-80A	Control	0.84 ± 0.03	2.86 ± 0.21	0.75 ± 0.06	3.81 ± 0.12
	1 week	0.87 ± 0.05	2.38 ± 0.19	0.88 ± 0.07	2.70 ± 0.16
	12 weeks	0.77 ± 0.03	2.10 ± 0.17	0.80 ± 0.07	2.63 ± 0.14
	24 weeks	0.85 ± 0.04	2.28 ± 0.17	0.80 ± 0.05	2.90 ± 0.10
*p*		0.07	0.04	0.19	0.03
Pel-80A	Control	0.94 ± 0.05	0.90 ± 0.07	0.88 ± 0.06	1.03 ± 0.08
	1 week	0.90 ± 0.05	0.91 ± 0.08	0.89 ± 0.07	1.03 ± 0.09
	12 weeks	0.88 ± 0.06	0.89 ± 0.08	0.87 ± 0.06	1.03 ± 0.08
	24 weeks	0.78 ± 0.05	0.87 ± 0.06	0.88 ± 0.06	0.99 ± 0.07
*p*		0.06	0.81	0.83	0.90

The data are shown as the mean value for three biological donors ± standard deviation.

**Table 3:** Dependence of the strength of the vascular grafts made of Tec-80A on the time of their functioning in the rat infrarenal position.

	Tensile Strength, MPa	Elongation, %	Thickness of the VG, µm
Initial	6.6 ± 0.6	308 ± 9	138 ± 9
3 months	4.8 ± 0.7	304 ± 10	170 ± 17
6 months	7.7 ± 0.7	290 ± 13	198 ± 16
*p*	0.03	0.39	0.04

The data are shown as the mean value for three biological donors ± standard deviation.

## References

[1] LaPorte R.J. (2017). Hydrophilic Polymer Coatings for Medical Devices.

[2] Kheradvar A., Groves E.M., Dasi L.P., Alavi S.H., Tranquillo R., Grande-Allen K.J., Simmons C.A., Griffith B., Falahatpisheh A., Goergen C.J. (2015). Emerging trends in heart valve engineering: Part I. Solutions for future. Ann. Biomed. Eng..

[3] Tatai L., Moore T.G., Adhikari R., Malherbe F., Jayasekara R., Griffiths I., Gunatillake P.A. (2007). Thermoplastic biodegradable polyurethanes: The effect of chain extender structure on properties and in-vitro degradation. Biomaterials.

[4] Hergenrother R.W., Wabers H.D., Cooper S.L. (1993). Effect of hard segment chemistry and strain on the stability of polyurethanes: In vivo biostability. Biomaterials.

[5] Szycher M. (1988). Biostability of polyurethane elastomers: A critical review. J. Biomater. Appl..

[6] Simmons A., Hyvarinen J., Odell R.A., Martin D.J., Gunatillake P.A., Noble K.R., Poole-Warren L.A. (2004). Long-term in vivo biostability of poly (dimethylsiloxane)/poly (hexamethylene oxide) mixed macrodiol-based polyurethane elastomers. Biomaterials.

[7] Grasl C., Bergmeister H., Stoiber M., Schima H., Weigel G. (2010). Electrospun polyurethane vascular grafts: In vitro mechanical behavior and endothelial adhesion molecule expression. J. Biomed. Mater. Res. A.

[8] Bergmeister H., Seyidova N., Schreiber C., Strobl M., Grasl C., Walter I., Messner B., Baudis S., Fröhlich S., Marchetti-Deschmann M. (2015). Biodegradable, thermoplastic polyurethane grafts for small diameter vascular replacements. Acta Biomater..

[9] Hasan A., Memic A., Annabi N., Hossain M., Paul A., Dokmeci M.R., Dehghani F., Khademhosseini A. (2014). Electrospun scaffolds for tissue engineering of vascular grafts. Acta Biomater..

[10] Xu C., Inai R., Kotaki M., Ramakrishna S. (2004). Electrospun nanofiber fabrication as synthetic extracellular matrix and its potential for vascular tissue engineering. Tissue Eng..

[11] Rocco K.A., Maxfield M.W., Best C.A., Dean E.W., Breuer C.K. (2014). In vivo applications of electrospun tissue-engineered vascular grafts: A review. Tissue Eng. Part B Rev..

[12] Chernonosova V.S., Kvon R.I., Stepanova A.O., Larichev Y.V., Karpenko A.A., Chelobanov B.P., Kiseleva E.V., Laktionov P.P. (2017). Human serum albumin in electrospun PCL fibers: Structure, release, and exposure on fiber surface. Polym. Adv. Technol..

[13] Jing X., Mi H.Y., Salick M.R., Cordie T.M., Peng X.F., Turng L.S. (2015). Electrospinning thermoplastic polyurethane/graphene oxide scaffolds for small diameter vascular graft applications. Mat. Sci. Eng. C Mater..

[14] Firoozi S., Derakhshan M.A., Karimi R., Rashti A., Negahdari B., Faridi Majidi R., Mashaghi S., Ghanbari H. (2017). Fabrication and characterization of nanofibrous tricuspid valve scaffold based on polyurethane for heart valve tissue engineering. Nanomed. Res. J..

[15] Puperi D.S., Kishan A., Punske Z.E., Wu Y., Cosgriff-Hernandez E., West J.L., Grande-Allen K.J. (2016). Electrospun polyurethane and hydrogel composite scaffolds as biomechanical mimics for aortic valve tissue engineering. ACS Biomater. Sci. Eng..

[16] D’Amore A., Luketich S.K., Raffa G.M., Olia S., Menallo G., Mazzola A., D’Accardi F., Grunberg T., Gu X., Pilato M. (2018). Heart valve scaffold fabrication: Bioinspired control of macro-scale morphology, mechanics and micro-structure. Biomaterials.

[17] Wang Y., Li P., Xiang P., Lu J., Yuan J., Shen J. (2016). Electrospun polyurethane/keratin/AgNP biocomposite mats for biocompatible and antibacterial wound dressings. J. Mater. Chem. B.

[18] Masaeli E., Karamali F., Loghmani S., Eslaminejad M.B., Nasr-Esfahani M.H. (2017). Bio-engineered electrospun nanofibrous membranes using cartilage extracellular matrix particles. J. Mater. Chem. B.

[19] Chernonosova V.S., Kvon R.I., Kiseleva E.V., Stepanova A.O., Laktionov P.P. (2017). The study of the surface layer of 3D-matrices for tissue engineering. Biochem. (Moscow) Suppl. Ser. B Biomed. Chem..

[20] Chernonosova V.S., Gostev A.A., Gao Y., Chesalov Y.A., Shutov A.V., Pokushalov E.A., Karpenko A.A., Laktionov P.P. (2018). Mechanical properties and biological behavior of 3D matrices produced by electrospinning from protein-enriched polyurethane. BioMed Res. Int..

[21] Chernonosova V.S., Gostev A.A., Chesalov Y.A., Karpenko A.A., Karaskov A.M., Laktionov P.P. (2018). Study of hemocompatibility and endothelial cell interaction of Tecoflex-based electrospun vascular grafts. Int. J. Polym. Mater. PO.

[22] Farah S., Kunduru K.R., Basu A., Domb A.J. (2015). Molecular weight determination of polyethylene terephthalate. Poly (Ethylene Terephthalate) Based Blends, Composites and Nanocomposites.

[23] Kolesov S.V., Zaidullin I.S., Spirikhin L.V., Volodina V.P., Kukovinets O.S., Sigaeva N.N., Vildanova R.R. (2016). Modification of chitosan and hyaluronic acid to obtain sustainable hydrogels. Physical Chemistry for the Chemical and Biochemical Sciences.

[24] Popova I.V., Stepanova A.O., Plotnikova T.A., Sergeevichev D.S., Akulov A.E., Pokushalov A.A., Laktionov P.P., Karpenko A.A. (2015). Study of patency of vascular grafts manufactured by means of electrospinning. Angiol. Sosud. Khir..

[25] (1998). ISO 7198:1998 Cardiovascular Implants—Tubular Vascular Prostheses.

[26] Kucińska-Lipka J., Gubańska I., Janik H. (2013). Gelatin-modified polyurethanes for soft tissue scaffold. Sci. World J..

[27] Gregory E.K., Webb A., Vercammen J.M., Kelly M.E., Akar B., van Lith R., Bahnson E.M., Jiang W., Ameer G.A., Kibbe M.R. (2018). Inhibiting intimal hyperplasia in prosthetic vascular grafts via immobilized all-trans retinoic acid. J. Control. Release.

[28] Kohler T.R., Kirkman T.R., Kraiss L.W., Zierler B.K., Clowes A.W. (1991). Increased blood flow inhibits neointimal hyperplasia in endothelialized vascular grafts. Circ. Res..

[29] Martins M.C., Wang D., Ji J., Feng L., Barbosa M.A. (2003). Albumin and fibrinogen adsorption on PU–PHEMA surfaces. Biomaterials.

[30] Stokes K.B. (1988). Polyether polyurethanes: Biostable or not?. J. Biomater. Appl..

[31] Gruendling T., Junkers T., Guilhaus M., Barner-Kowollik C. (2010). Mark–Houwink parameters for the universal calibration of acrylate, methacrylate and vinyl acetate polymers determined by online size-exclusion chromatography—Mass spectrometry. Macromol. Chem. Phys..

[32] Ma J., Liang B., Cui P., Dai H., Huang R. (2003). Dilute solution properties of hydrophobically associating polyacrylamide: Fitted by different equations. Polymer.

[33] Gunatillake P.A., Martin D.J., Meijs G.F., McCarthy S.J., Adhikari R. (2003). Designing biostable polyurethane elastomers for biomedical implants. Aust. J. Chem..

[34] Pinchuk L. (1995). A review of the biostability and carcinogenicity of polyurethanes in medicine and the new generation of ‘biostable’ polyurethanes. J. Biomater. Sci. Polym. Ed..

[35] Gostev A.A., Karpenko A.A., Laktionov P.P. (2018). Polyurethanes in cardiovascular prosthetics. Polym. Bull..

[36] Mathur A.B., Collier T.O., Kao W.J., Wiggins M., Schubert M.A., Hiltner A., Anderson J.M. (1997). In vivo biocompatibility and biostability of modified polyurethanes. J. Biomed. Mater. Res..

[37] Schubert M.A., Wiggins M.J., Anderson J.M., Hiltner A. (1997). Comparison of two antioxidants for poly (etherurethane urea) in an accelerated in vitro biodegradation system. J. Biomed. Mater. Res..

[38] Thomas V., Jayabalan M. (2009). A new generation of high flex life polyurethane urea for polymer heart valve—Studies on in vivo biocompatibility and biodurability. J. Biomed. Mater. Res. A.

[39] McCarthy S.J., Meijs G.F., Mitchell N., Gunatillake P.A., Schindhelm K. (1997). In-vivo degradation of polyurethanes: Transmission-FTIR microscopic characterization of polyurethanes sectioned by cryomicrotomy. Biomaterials.

[40] Therona J.P., Knoetzeb J.H., Sandersonc R.D., Hunterd R., Mequaninte K., Franza T., Zillaa P., Bezuidenhouta D. (2010). Modification, crosslinking and reactive electrospinning of a thermoplastic medical polyurethane for vascular graft applications. Acta Biomater..

[41] Bergmeister H., Schreiber C., Grasl C., Walter I., Plasenzotti R., Stoiber M., Bernhard D., Schima H. (2013). Healing characteristics of electrospun polyurethane grafts with various porosities. Acta Biomater..

