# Correction: Gostev et al. In Vivo Stability of Polyurethane-Based Electrospun Vascular Grafts in Terms of Chemistry and Mechanics. *Polymers* 2020, *12*, 845

**DOI:** 10.3390/polym14112263

**Published:** 2022-06-01

**Authors:** Alexander A. Gostev, Inna K. Shundrina, Vitaliy I. Pastukhov, Alexey V. Shutov, Vera S. Chernonosova, Andrey A. Karpenko, Pavel P. Laktionov

**Affiliations:** 1 Meshalkin National Medical Research Center, Ministry of Health of the Russian Federation, 630055 Novosibirsk, Russia; andreikarpenko@rambler.ru (A.A.K.); lakt@niboch.nsc.ru (P.P.L.); 2 Vorozhtsov Novosibirsk Institute of Organic Chemistry, Siberian Branch, Russian Academy of Sciences, 630090 Novosibirsk, Russia; i.shundrina@nsu.ru (I.K.S.); v.pastukhov@nsu.ru (V.I.P.); 3 Novosibirsk State University, ul. Pirogova, 2, 630090 Novosibirsk, Russia; a.shutov@g.nsu.ru; 4 Institute of Chemical Biology and Fundamental Medicine, Siberian Branch, Russian Academy of Sciences, 630090 Novosibirsk, Russia; vera_mal@niboch.nsc.ru; 5 Lavrentiev Institute of Hydrodynamics, Siberian Branch, Russian Academy of Sciences, 630090 Novosibirsk, Russia

The authors wish to make a change to the published paper [1]. In the original manuscript, the authors made a mistake in Figure 1. The tecoflex graft image in the first week of observation moved to the place of the pellethane graft image at the 12th week of observation. Additionally, the image of the pellethane graft at the 12th week of observation moved to the place of the image of the tecoflex graft in the first week of observation. The corrected Figure 1 is presented below. 

The authors apologize for any inconvenience caused, and the change does not affect the scientific results. The manuscript will be updated.

## Figures and Tables

**Figure 1:**
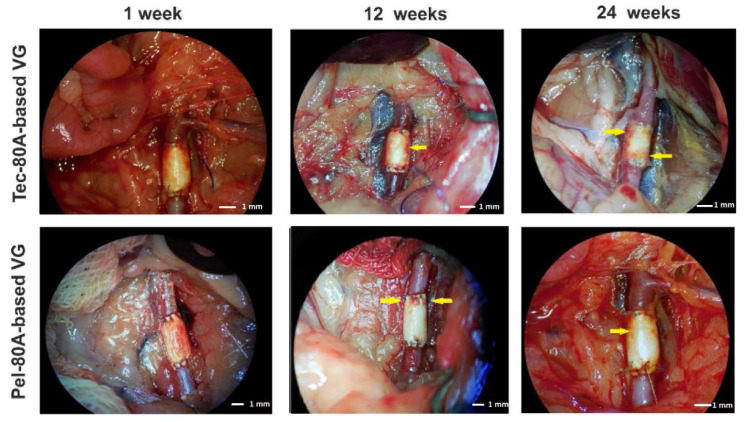
The vascular graft (VG) view during explantation at different points of observation (Carl Zeiss OPMI Pico surgical microscope). The arrows demonstrate the ingrowth of tissues from the outer VG side.
